# High Interaction Variability of the Bivalve-Killing Dinoflagellate *Heterocapsa circularisquama* Strains and Their Single-Stranded RNA Virus HcRNAV Isolates

**DOI:** 10.1264/jsme2.ME12106

**Published:** 2012-12-27

**Authors:** Natsuko Nakayama, Akihiro Fujimoto, Hisae Kawami, Yuji Tomaru, Naotsugu Hata, Keizo Nagasaki

**Affiliations:** 1 National Research Institute of Fisheries and Environment of Inland Sea, Fisheries Research Agency, 2–17–5 Maruishi, Hatsukaichi, Hiroshima 739–0452, Japan; 2 Hiroshima University, Higashi-Hiroshima 980–0813 Japan; 3 Mie Prefecture Fisheries Research Institute, Shima, Mie 517–0404, Japan

**Keywords:** HcRNAV, *Heterocapsa circularisquama*, clonal variation, major capsid protein, host-virus system

## Abstract

HcRNAV is a single-stranded RNA (ssRNA) virus that specifically infects the bivalve-killing dinoflagellate, *Heterocapsa circularisquama*. HcRNAV strains are grouped into 2 types (UA and CY), based on intra-species host specificity and the amino acid sequence of the major capsid protein (MCP). In the present study, we report the isolation of novel HcRNAV clones (*n*=51) lytic to the *H. circularisquama* strains, HU9433-P, HCLG-1, 05HC05 and 05HC06. HcRNAV34, HcRNAV109, HcRNAV641, and HcRNAV659, which displayed lytic activity against the strains, HU9433-P, HCLG-1, 05HC05, and 05HC06, respectively, were selected as typical virus clones and were intensively examined. The infection intensity of each host-virus combination was analyzed by examining the algicidal activity, detecting the intracellular replication of the viral RNA as well as the appearance of host cells with a morphologically abnormal nucleus post-infection. Interestingly, the strains, 05HC05 and 05HC06, were markedly sensitive to HcRNAV641 and HcRNAV659, respectively. Tertiary structural modeling predicted 4 unique amino acid (aa) substitutions in HcRNAV659-MCP to be exposed to an ambient water environment, which contributed towards determining its infection specificity. Neighbor-joining analysis of MCP aa sequences from HcRNAV clones revealed 3 clades, namely, the CY type and the UA1 and UA2 subtypes. The HcRNAV clones lytic to HCLG-1 (ex. HcRNAV109), HU9433-P and 05HC05 (ex. HcRNAV34), and 05HC06 (ex. HcRNAV659) were categorized into CY type, UA1 and UA2 subtypes, respectively. The present study highlights the complexity of the *H. circularisquama*-HcRNAV host-virus system, i.e., clonal variation, microbial control, and ecology in a natural algal population.

The marine dinoflagellate, *Heterocapsa circularisquama* Horiguchi, is one of the most harmful red-tide bloom-forming microalgae, which specifically kills shellfish (2, 6, 7). HcRNAV, a small icosahedral virus that specifically infects *H. circularisquama*, harbors a linear single-stranded RNA (ssRNA) genome of approximately 4.4 kb in length (18). The HcRNAV genome contains 2 open reading frames (ORFs), wherein the upstream ORF-1 encodes a replication-related polyprotein, and the downstream ORF-2 codes for a major capsid protein (MCP) of approximately 38 kDa (11). On the basis of our previous field surveys, HcRNAV has been regarded as one of the key components that determine the population dynamics of *H. circularisquama* in the natural environment (12, 17). In our previous study, we performed several combinations of cross-reactivity tests between *H. circularisquama* strains and HcRNAV clones collected from different locations in western Japan over a period of time, and showed that HcRNAV has at least 2 distinct ecotypes (types UA and CY) that exhibit complementary intra-species host specificity (18). Furthermore, the tertiary structure of the capsid proteins of HcRNAV34 and HcRNAV109, predicted based on the deduced amino acid sequence of the MCP, indicated that intra-species host specificity of HcR-NAV is determined by the nanostructures on the virus surface exposed to the ambient water environment and that these exterior structures might affect binding to suitable host cells (11). For several years, this reciprocal relationship has been considered to be the principal mechanism of interaction between the host and the virus. Later, Mizumoto *et al.* characterized 2 *H. circularisquama* strains (05HC05 and 05HC06, isolated from Ago Bay in 2005), which did not show any apparent virus-induced cell lysis when inoculated with either HcRNAV34 or HcRNAV109 (10). In particular, strain 05HC06 showed marked resistance to both the HcRNAV strains, which was confirmed by the absence of detectable HcRNAV genome replication in northern hybridization analysis. These results showed that *H. circularisquama* strains exhibit diverse responses to infection by HcRNAV clones, and further suggested the possible existence of unknown host-virus combinations in natural environments.

In view of the above, in the present study, HcRNAV clones showing lytic activity to the *H. circularisquama* strains, 05HC05 and/or 05HC06, were successfully isolated. Further, we analyzed intra-species host specificity by examining the host response to inoculation of the isolated HcRNAV clone, detecting the intracellular replication of viral RNA, and the appearance of host cells with a morphologically abnormal nucleus post-infection (19). Furthermore, we predicted the tertiary structure of HcRNAV MCP and determined the phylogenetic diversity based on the amino acid sequence of the MCP core region of the 4 typical HcRNAV clones isolated by using different *H. circularisquama* strains as hosts. Through comparative analyses, we also elucidated the highly complex host-virus dynamics in natural environments.

## Materials and Methods

### Hosts and Viruses

Four strains of *H. circularisquama*, HU9433-P (isolated from Uranouchi Bay, Kochi Prefecture, Japan), HCLG-1 (Gokasho Bay, Mie Prefecture, Japan), 05HC05, and 05HC06 (Ago Bay, Mie Prefecture, Japan) were used in this study (10, 18). In our previous experiments, the latter 2 strains showed high resistance to the most extensively studied HcRNAV clones, HcRNAV34 and HcRNAV109 (11, 18). Algal cell cultures were grown in modified SWM3 medium enriched with 2 nM Na_2_SeO_3_ (1, 3, 4), and incubated under an alternating 12-h light–dark cycle; the light (130 to 150 μmol of photons m^−2^ s^−1^) was provided by cool white fluorescent illumination at 20°C. The HcRNAV clones obtained in this fashion were then screened for lysis, according to the method described by Tomaru *et al.* (18). This led to the successful isolation of a number of HcRNAV clones infecting each host strain. In the present study, 53 HcRNAV clones were examined (Table 1). In particular, the relationship between the 4 typical HcRNAV clones (HcRNAV-34, -109, -641, and -659) and their host strains was intensively characterized in this study. The viruses were cryopreserved at −20°C without the addition of any cryoprotectant until further use.

### Cross reactivity tests

Sixteen combinations of cross-reactivity tests were performed between the 4 *H. circularisquama* strains and the corresponding HcRNAV clones. Briefly, an exponentially growing culture of *H. circularisquama* (40 mL) was inoculated with 1 mL of virus suspension at a multiplicity of infection of *ca.* 100–500, and incubated under the conditions described in the previous section. Host cultures inoculated with sterilized SWM3 medium served as controls. An aliquot of each culture was sampled every 24 h for 6 days.

### Host cell counts

The abundance of *H. circularisquama* cells was measured immediately after sampling, without fixation, using an Epics XL-MCL flow cytometer (Beckman Coulter, Fullerton, CA, USA) equipped with a 488-nm air-cooled argon ion laser (15 mW) and standard filters. Fluorescent microspheres (Flow-Count, 10 μm; Beckman Coulter) were added as an internal reference. Readings were collected for 1 min in logarithmic mode and were analyzed using EXPO32ADC software (Beckman Coulter).

### Northern blot analysis

A decline in host cell number was observed in the samples collected at 0, 1, 2 and 3 days post-inoculation (dpi) and the samples were further analyzed by northern hybridization. An aliquot of cell suspension was removed from the culture centrifuged at 13,000×*g* for 3 min, and the resultant pellets were stored at −80°C until RNA extraction. The frozen cells were homogenized with plastic pestles in 100 μL RNase-free water and 40 μL RNA extraction buffer (5). The aqueous phase was extracted using phenol prior to ethanol precipitation. Northern blot analysis of the purified RNA was performed as previously described (9, 10). Briefly, viral negative-stranded RNA-specific digoxigenin-labeled RNA probes were transcribed from the previously constructed plasmid, pBSSK +MCP (9), and the resultant RNA signals were detected using a luminescence image analyzer (LAS-3000 mini; Fuji Photo Film, Tokyo, Japan). Based on the signals, the intracellular viral propagation for each host-virus combination was determined.

Furthermore, *H. circularisquama* cells sampled every 24 h for 6 days were fixed with 1% glutaraldehyde (final conc.) and maintained at 4°C. The cells were then stained with SYBR-GOLD (Molecular Probes, Eugene, OR, USA) according to the methods described by Tomaru *et al.* (19). More than 300 cells were observed under fluorescence microscopy and the proportion of cells that had a morphologically abnormal nucleus was evaluated. Cells without virus inoculation served as negative controls. In this analysis, when an abnormal nucleus was observed in more than 50% and 10% of *H. circularisquama* cells, viral lytic activity was labeled as “sensitive.”

### Tertiary structure prediction of HcRNAV MCP

Based on the results of our previous study, the *H. circularisquama* strain, 05HC06, which exhibited high resistance upon infection with the clones HcRNAV34 and HcRNAV109, showed apparent cell lysis when inoculated with HcRNAV659. Therefore, the genomic RNA of the HcRNAV659 clone was purified, and its ORF-2 (MCP gene) was completely sequenced according to the method described by Nagasaki *et al.* (11). Subsequently, based on the deduced amino acid sequence, the tertiary structure of HcRNAV659 MCP was predicted *in silico* using a Protein Discovery Full Automatic Modeling System (PDFAMS; In-Silico Sciences, Japan) (http://pd-fams.com/index.html). The results were manually modified, and the HcRNAV659 model was compared against those of HcRNAV34 and HcRNAV109, as previously reported by Nagasaki *et al.* (11).

The structural data were viewed using the RasMol 2.6 Beta-2a program (http://www.umass.edu/microbio/rasmol/getras.htm).

### Reverse transcription PCR (RT-PCR) of the MCP gene fragment

Genomic RNAs of the tested HcRNAV clones (Table 1) were purified according to the method described in the previous section. The purified RNAs were reverse-transcribed to obtain cDNAs by using random primers and the cDNA synthesis kit with SuperscriptIII Reverse Transcriptase (Invitrogen, Carlsbad, CA, USA), according to the manufacturer’s recommendations. PCR was designed to amplify the fragments of HcRNAV ORF-2 encoding the MCP, which spanned 3 of the 4 variable regions containing amino acid substitutions that were found to result in amino acid differences between HcRNAV34 and HcRNAV109. PCR was performed with a GeneAmp PCR System 9700 thermal cycler (Applied Biosystems, Carlsbad, CA, USA) using the forward and reverse primers, ORF2-A1: 5′-TTTCACCCTGAGCACCTTCCG C-3′ and ORF2-B1: 5′-CGCCATGCAACGCATTAAGCAGC-3′, respectively, which were designed based on the nucleotide sequences of HcRNAV34 and HcRNAV109 (11).

The total volume of the reaction mixture was 20 μL, containing 1 μL of each primer (10 μM each), 2 μL of 2 mM dNTPs, 2 μL of 10× buffer, 0.2 μL of Blend Taq (Toyobo, Osaka, Japan), and 2 μL of cDNA as a template. PCR conditions were as follows: initial denaturation at 94°C for 4 min followed by 30 cycles of denaturation at 94°C for 30 s, annealing at 55°C for 5 s, extension at 72°C for 1 min, and final extension at 72°C for 5 min. The resultant products were electrophoresed in 2% (w/v) agarose S gels (Nippon Gene). The nucleic acids were visualized by ethidium bromide staining.

### Phylogenetic analysis of HcRNAV MCP amino acid sequence

The PCR products were purified using an ExoSAP-IT kit (GE Healthcare, Little Chalfont, UK), and directly sequenced in an ABI PRISM 3100 Genetic Analyzer (Applied Biosystems) using the BigDye Terminator v3.1 Cycle Sequencing Kit (Applied Biosystems), and the primers ORF2-A1 and ORF2-B1. Each nucleotide sequence was translated into an amino acid sequence using the universal genetic code. The deduced amino acid sequences were then aligned, and a phylogenetic tree was constructed with the Clustal W program using the neighbor-joining method (NJ) (15) and NJ-plot software (13), with which 1,000-fold bootstrap analysis was carried out to test the reliability of the NJ tree.

## Results

### Infection specificity of HcRNAV clones

By employing seawater and sediment samples collected from the western coast of Japan, HcRNAV clones (*n*) lytic to *H. circularisquama* strains, HU9433-P (*n*=21), HCLG-1 (*n*=9), 05HC05 (*n*=9), and 05HC06 (*n*=12) were successfully isolated (Table 1). The lengths of the genomes of the isolated HcRNAV clones (*n*=51) were in close agreement with the ORF-2 genome size (1.08-kb) for HcRNAV34 and HcRNAV109, predicted using denaturing gel electrophoresis. HcRNAV34 (lytic to HU9433-P), HcRNAV109 (lytic to HCLG-1), HcRNAV641 (lytic to 05HC05), and HcRNAV659 (lytic to 05HC06) were selected as typical virus clones and were intensively examined in the subsequent experiments.

**Fig. 1:**
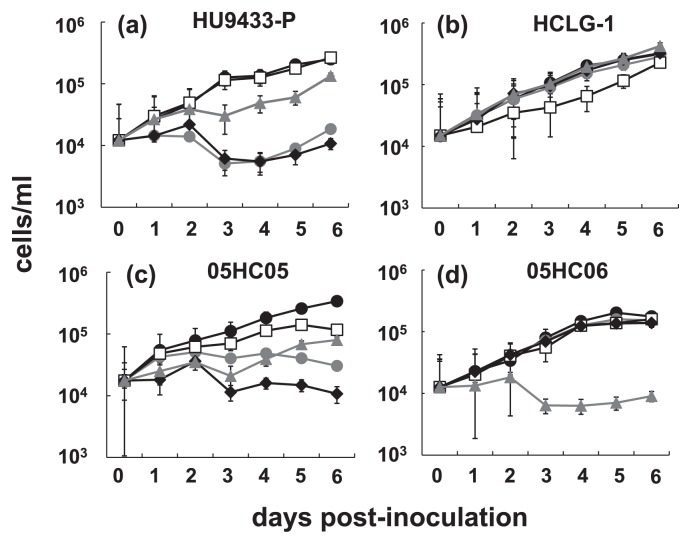
Changes in the abundance of *Heterocapsa circularisquama* cells after inoculation with HcRNAV. Algal cells were measured using flow cytometry (refer “Materials and Methods” section). Algal host strains HU9433-P (a), HCLG-1 (b), 05HC05 (c), and 05HC06 (d) were inoculated with HcRNAV 34 ( 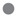), HcRNAV109 (□), HcRNAV641 (◆), and HcRNAV 659 ( 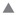) on day 0. In this study, algal cultures inoculated with sterilized SWM3 served as controls (●).

The 4 HcRNAV clones were inoculated into their host cultures, and the resultant viral lysates were used as the inocula. The infection specificity of the 4 typical HcRNAV clones and the susceptibility of hosts to the isolated viruses were tested by adding lysate to the 4 exponentially growing strains of *H. circularisquama. H. circularisquama* HU9433-P was markedly sensitive to both HcRNAV34 and HcRNAV641, slightly sensitive to HcRNAV659, but resistant to HcRNAV109 (Fig. 1a). In contrast, *H. circularisquama* HCLG-1 was susceptible only to HcRNAV109, and resistant to the other 3 HcRNAV clones (Fig. 1b). Notably, *H. circularisquama* 05HC05 was highly sensitive to HcRNAV641 and less sensitive to both HcRNAV34 as well as HcRNAV659, while being resistant to HcRNAV109 (Fig. 1c). The notable host strain *H. circularisquama* 05HC06 was sensitive only to HcRNAV659, with the other 3 virus clones not showing any lytic activity against 05HC06. Our findings are in agreement with the results reported by Mizumoto *et al.*, who showed that *H. circularisquama* 05HC06 demonstrated high resistance to the clones, HcRNAV34 and HcRNAV109 (10).

**Fig. 2:**
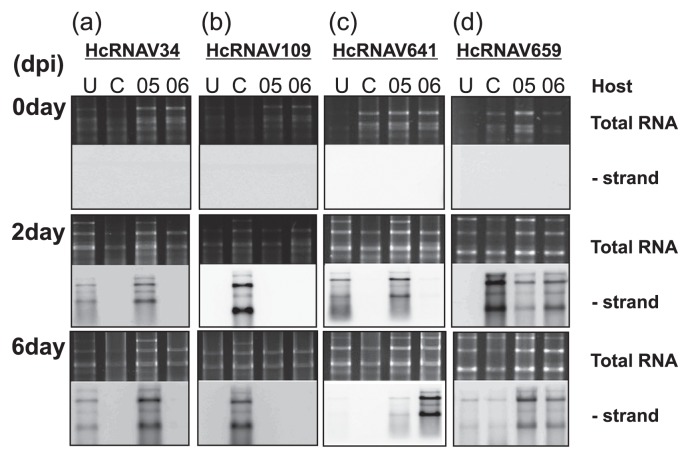
Accumulation of negative-stranded RNA of HcRNAV; (a) HcRNAV 34, (b) HcRNAV109, (c) HcRNAV641, and (d) HcRNAV659 in *H. circularisquama* strains HU9433-P (U), HCLG-1 (C), 05HC05 (05), and 05HC06 (06), respectively. The terms U, C, 05, and 06 indicate *H. circularisquama* strains HU9433-P, HCLG-1, 05HC05, and 05HC06, respectively. The 0 day, 2 days, and 6 days indicate the day post-inoculation (dpi). Total RNA was extracted from *H. circularisquama* cells, separated by gel electrophoresis (upper photographs), and blotted onto the membranes. Then, the total RNA was subjected to northern hybridization analysis (lower photographs).

In parallel, the intra-species specificity of each typical HcRNAV clone was examined using the northern hybridization method, where detection of the band corresponding to the negative-strand RNA of HcRNAV indicated the occurrence of intracellular replication (Fig. 2). The replication of HcRNAV34 was detected in *H. circularisquama* strains, HU9433-P (infected by UA-type HcRNAVs) and 05HC05, but not in the other 2 host strains (Fig. 2a). In contrast, the replication of HcRNAV109 was detected only in the original host strain HCLG-1 (infected by CY-type HcRNAVs), as expected, but not in the remaining 3 host strains (Fig. 2b). With the HcRNAV641 clone, replication was detected in HU9433-P, 05HC05, and 05HC06 at 2 dpi, 2 dpi, and 6 dpi, respectively (Fig. 2c). Interestingly, the replication of HcRNAV641 and HcRNAV659 was detected to different degrees in all 4 host strains. For instance, the accumulation of negative-stranded HcRNAV659 RNAs was observed at 2 and/or 6 dpi in a few host-virus combinations (Fig. 2d). It was reasonable that a strong band was observed at 2 dpi for the combination of HcRNAV659 and 05HC06; however, with the combination of HcRNAV659 and HCLG-1, the signal was found to be unexpectedly intense. Notably, in the combination of HcRNAV641 and 05HC06, a strong band was observed only at 6 dpi.

**Fig. 3:**
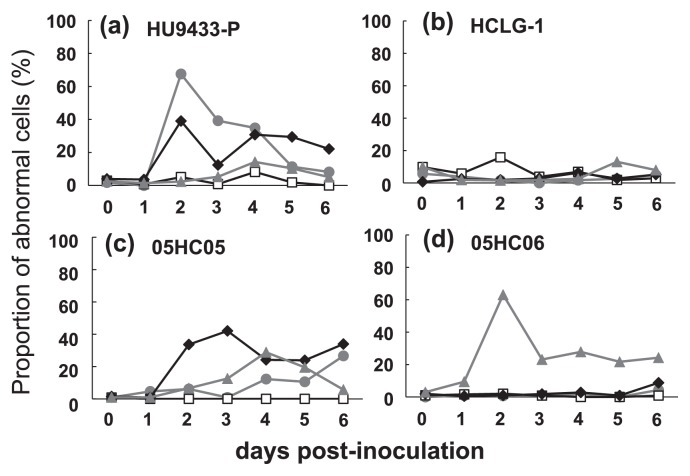
Changes in the proportion of cells with abnormal nuclei in *H. circularisquama* HU9433-P (a), HCLG-1 (b), 05HC05 (c), and 05HC06 (d), which had an abnormal nucleus after inoculation of HcR-NAV 34 ( 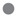), HcRNAV109 (□), HcRNAV641 (◆), and HcRNAV 659 ( 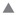).

Furthermore, the proportion of host cells containing an abnormal nucleus was estimated as an index of viral lytic activity (Fig. 3). Using this analysis, HcRNAV34 and HcRNAV641 were shown to be lytic to *H. circularisquama* strains HU9433-P and 05HC05. This data supported the results of the inoculation experiments and northern hybridization described in the previous sections of this study (Fig. 3a, c). The lytic activity of HcRNAV109 was detected only in HCLG-1, although there was no significant increase in the proportion of cells that had an abnormal nucleus (Fig. 3b). In contrast to other clones, HcRNAV659 displayed strong lytic activity against its original host strain, 05HC06, when compared to the other 3 host strains (Fig. 3d).

### Tertiary structure prediction of HcRNAV659 MCP

**Fig. 4:**
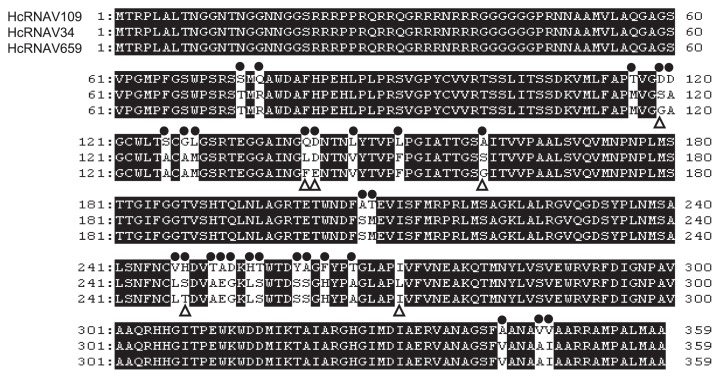
Complete amino acid alignment of the MCP of the typical virus clones tested in the present study (HcRNAV 109, HcRNAV34, and HcRNA659). Closed circles indicate the amino acid residue site differing between HcRNAV109 and HcRNAV659; open triangles indicate the amino acid residue site differing between HcRNAV34 and HcRNAV659. (Note that there was no substitution in the core region of MCP gene from the clones, HcRNAV34 and HcRNAV641).

To determine whether the newly isolated virus clone, HcRNAV659 differed significantly with respect to intra-species specificity when compared to the clones HcRNAV34 (UA-type) and HcRNAV109 (CY-type), the tertiary structure of its MCP was predicted *in silico* (Fig. S1). By aligning the MCP amino acid sequence of HcRNAV659 against those of the clones HcRNAV34 and HcRNAV109, the numbers of substituted amino acid residues were found to be 6 and 29, respectively (Fig. 4). Upon analyzing the *in silico*-predicted model of HcRNAV659 MCP, it was noted that a few of the amino acid substitutions were located on the outside of the capsid protein (exposed to the ambient water environment). In contrast, no amino acid substitution was found on the reverse (interior) of the capsid protein (Fig. S1).

### Phylogenetic analysis

**Fig. 5:**
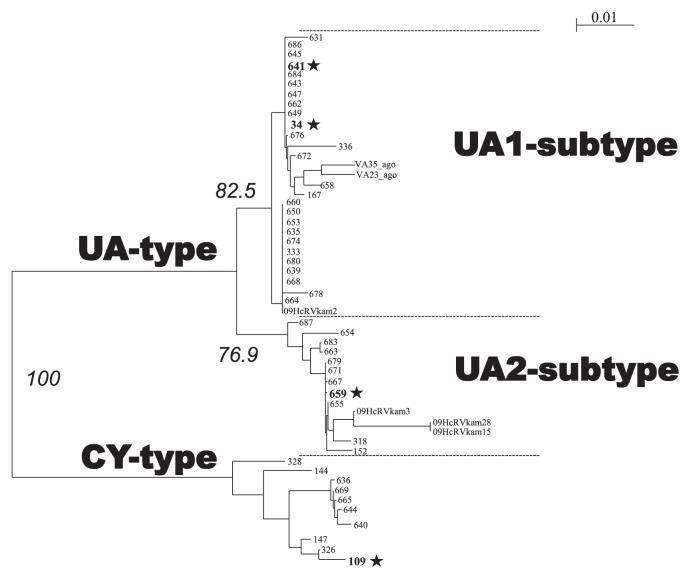
Phylogenetic tree constructed from the deduced amino acid sequences encoded by the major capsid protein gene ORF-2 fragment in various HcRNAV strains. *symbols indicate typical virus clones in this study. Nodes with bootstrap values (in italics) below 70% were collapsed. Scale bar represents the frequency of the occurrence of amino acid substitutions per residue.

Phylogenetic analysis was performed to investigate the relationship between the novel HcRNAV isolates. A phylo-genetic tree was constructed based on the deduced amino acid sequences of HcRNAV MCP encoded by ORF-2, which also included the previously reported sequence data (Table 1, Fig. 5) (11). Consequently, all the virus clones that showed lytic activity against HCLG-1 were grouped into a single phylogenetic cluster supported by the 100% bootstrap value, and hence labeled as “CY type.” In contrast, the virus clones lytic to HU9433-P, 05HC05, or 05HC06 strains were grouped into a “UA-type” cluster, which was further stratified into 2 sub-clusters (Fig. 5). Notably, the virus clones isolated by using 05HC06 as the host strain were all categorized into 1 of the 2 sub-clusters within the UA type, which was moderately supported by a bootstrap value of ~77%. This sub-cluster was designated as the “UA2 subtype” (Fig. 5). The remaining clones isolated with either HU9433-P or 05HC05 strains were grouped into the “UA1-subtype” sub-cluster supported by a bootstrap value of ~83% (Fig. 5). Hence, the amino acid sequences of the MCP fragments were grouped into 3 phylogenetic clusters.

## Discussion

### Intra-species specificity of HcRNAV

In this study, the infection intensity of each host-virus combination was analyzed by examining the algicidal activity, detecting the intracellular replication of the viral RNA as well as the changes associated with the number of abnormal host cells post-infection. The results obtained were similar for most of the combinations, with the exception of a few (Table 2). Consistent with the results of a previous study, which categorized HcRNAV109 and HcRNAV34 into CY and UA types, the present study demonstrated that HcRNAV109 displayed the most distinctive pattern of infection. The infectivity pattern of HcRNAV659 isolated using 05HC06 strain was strikingly different from that of HcRNAV34 and HcRNAV 109, which lends supports to the observation that 05HC06 strain exhibited resistance to the clones HcRNAV34 and HcRNAV 109 (9, 11). Earlier studies demonstrated that the intra-species specificity of HcRNAV34 and HcRNAV109 was determined by the nanostructures present on the exterior surface of the capsid. The present study attributed the differences with respect to intra-species specificity between HcRNAV659 and that of HcRNAV34 and HcRNAV109 more specifically to the presence of typical amino acid substitutions present on the exterior side of the capsid protein sequence which is exposed to ambient water environment (Fig. 4). Despite the differences in their lytic activity against 05HC06 strain, the amino acid substitutions in the case of the HcRNAV659 clone were detected only in 6 residues, all of which are located in “hot spots” of the MCP sequence (Fig. 4), unlike HcRNAV34 clone, which harbored 29 substitutions. For instance, there were 4 unique substitutions in HcRNAV659 MCP (*i.e.*, Gly119, Phe141, Gly160, and Thr248), which significantly differed amongst the other 2 typical HcRNAV clones HcRNAV 34 and HcRNAV 109 (Fig. 4). Taken together, our results suggest that these amino acid residues might be linked to the determination of intra-species host specificity; therefore, our study highlights the mechanism underlying the binding specificity of the virus clone to suitable host cells; however, it should be further investigated from the perspective of structural biology.

### Variable response patterns of *H. circularisquama* to HcRNAV infection

In accordance with previous studies which reported the re-growth of eukaryotic microalgae following viral lysis, in the present study, virus-induced cell lysis in some *H. circularisquama* strains was followed by cell growth upon inoculation (Fig. 1) (16, 20). A study by Tomaru *et al.* (19) demonstrated that the re-growth of *H. circularisquama* cells occurs in the presence of HcRNAV viruses in the cultures and showed that viral RNA replication does not occur in survivor cells physically transfected with viral RNA, indicating that the host cells rapidly change upon infection. However, it is still unclear whether only a very small proportion of the host cells in the culture harbored the virus resistance genes or acquired the defense mechanism following inoculation.

In all three experiments conducted in the current study, the results obtained for HcRNAV659 and HcRNAV641 were notably inconsistent within the measurement data (Table 2). The growth of *H. circularisquama* cells was not affected in the HCLG-1 culture inoculated with HcRNAV659 or in the 05HC06 culture inoculated with HcRNAV641 (Fig. 1b, d); however, accumulation of viral negative-stranded RNA (Fig. 2c, d) along with a moderate increase in the percentage of abnormal cells was observed at 2–6 dpi (Fig. 3b, d). Although HcRNAV659 did not cause any growth inhibition of HCLG-1 (Fig. 1d), replication of negative-strand RNA at 2dpi (Fig. 2d) and a moderate increase in the proportion of abnormal cells were detected (Fig. 3b). Another similar case was observed between HcRNAV641 and 05HC06 (Table 2). When *H. circularisquama* strain 05HC06 was inoculated with HcRNAV641, there was also no apparent decline in cell number until 6 dpi (Fig. 1d); nevertheless, as shown in Fig. 2c, accumulation of viral negative-stranded RNA along with a slight increase in the percentage of abnormal cells was observed at 6 dpi (Fig. 3d). In the previous study, the accumulation of viral negative-stranded RNA was clearly demonstrated at 1–2 dpi when virus-induced cell lysis was observed, following the inoculation of HCLG-1 with HcRNAV109 and HU9433-P with HcRNAV34 (10). In addition, Mizumoto *et al.* (10) demonstrated that *H. circularisquama* strains display diverse response patterns to HcRNAV inoculation, namely, sensitive, delayed lysis and resistant. Taking this into consideration, strain 05HC06 is permissive for intracellular replication of HcRNAV641, wherein the accumulation of viral negative-stranded RNA was clearly observed at 6 dpi (Fig. 2c), which indicated that the infectivity type of HcRNAV641 differs from both the sensitive and resistant responses and hence is a “delayed-lysis” variant. These responses thus promote the diversification of *H. circularisquama* strains and further indicate the existence of enormous variability in the infective specificity of HcRNAVs to *H. circularisquama* in natural marine environments.

To date, the detailed mechanism underlying the pathogenicity of HcRNAV infection has not been elucidated. The HcRNAV virion has been shown to be composed of 180 ORF-2 products, *i.e.*, identical capsid proteins (8); thus, it is unlikely that the HcRNAV virion possesses any special mechanism to inject its viral RNA into the host cell. An alternative possible entry route is the endocytosis of HcRNAV into the food vacuole of the host cell, which is followed by partial digestion due to lysosomal enzymes and subsequent decapsidation (exposure of viral RNA to cytosol). Consequently, HcRNAV genomic (+) RNA might be transcribed to (−) RNA, which functions as a “template” of the newly generated viral genome, *i.e.*, (+) RNA. Although the factors determining the replication efficiency have not been determined yet, it is likely that the intensity of the negative-stranded RNA signals might correspond to the efficiency of viral replication in the host cell. Further lending support to this hypothesis, our results showed the presence of strong signals in host-virus combinations where, the virus clone was isolated using the corresponding host strain. In theory, in every host-virus combination there exists a high binding affinity of the virus to the specific host and host-virus infection efficiency (whether it involves intensive lysis, moderate lysis, faint lysis, chronic infection, or resistance). Nanostructural differences of the MCP, in addition to the replication-related proteins and the 3′-stem-loop structure, might contribute to determining the infection efficiency of the virus; however, future studies elucidating the detailed molecular mechanism underlying the pathogenesis of HcR-NAV infection are warranted. Nevertheless, our results indicate that every viral clone-host strain combination has its own outcome, and that this diversity might influence the stagnation and succession of *H. circularisquama* populations in nature.

### Phylogeny of HcRNAV clones based on the MCP sequence

In a previous study by Tomaru *et al.*, HcRNAV clones were roughly grouped into 2 types (types, UA and CY) based on intra-species host specificity and the MCP amino acid sequences (18). Despite the isolation of 51 novel HcRNAV clones, the results of the current study strongly support the monophyly of the CY-type virus. Corroborating this, in the present study, the HcRNAV clones isolated using HU9433-P, 05HC05, or 05HC06 as hosts were grouped into a different phylogenetic cluster, within which 2 sub-clusters (UA1- and UA2-subtypes) were identified, which were further supported by moderate bootstrap values. While the UA1 subtype comprised of clones infectious to HU9433-P and 05HC05 strains, the clones isolated using HU9433-P and 05HC06 as hosts were categorized into the UA2 subtype. Notably, all the HcRNAV clones isolated using strain 05HC06 as the host belonged to the UA2 subtype. In comparison, those isolated with 05HC05 strain were categorized into the UA1 subtype; however, clones HcRNAV641 and HcRNAV659, which were isolated using 05HC05 and 05HC06 strains, respectively, were infectious to HU9433-P and, hence, the clones isolated with HU9433-P strain should logically be included in both UA1 and UA2 subtypes. Therefore, the HcRNAV clones tested in the present study were technically grouped into 3 clades (CY type, and UA1 and UA2 subtypes), indicating that there is potential for considerable diversity amongst the HcRNAV clones. Further strengthening these results, in our study, the ORF2 amino acid sequences of the HcRNAVs were grouped into 3 clusters widely spread over the phylogenetic tree irrespective of the place and time of the isolation of HcRNAVs (Table 1). Collectively, these results confirm that the *H. circularisquama* population in a particular environmental niche comprises highly diverse members.

Hence, the natural population of HcRNAV and its host *H. circularisquama* is likely more diverse than previously speculated (18), and the frequent algal bloom in a particular season or the persistent red tide may depend on the highly complex host-virus interaction.

### Implications

The virus replication rate is probably determined by the efficiency of RNA replication and/or viral entry into the host cell, which is further associated strongly with host mortality rates. The present study showed that the nanostructure of the HcRNAV virion surface may be highly diverse amongst the clones, and determines the intra-species host specificity of each clone. Under laboratory conditions, it is possible to infer the host-virus relationship in detail at the clonal level; however, it is much more complicated to obtain insight or actionable information regarding the natural populations (14). Hence, it is essential to assimilate the basic field data as well as the clonal variation data to accomplish scientific advances in this field of research.

When conducting this type of survey, it should be noted that researchers tend to focus their efforts (labor, time, and money) on host-virus combinations with which marked cell lysis is observed, whether intentionally or not, to obtain novel results that are easy to interpret. Consequently, there is the risk of a novel finding being misinterpreted as typical. Hence, the processes that occur in natural waters may be completely different from those observed in intensively studied “special” cases; therefore, it is important to recognize the diversity of host-virus systems in nature and the necessity of realistic as well as objective interpretations of the obtained experimental results.

## Supplementary Material



## Figures and Tables

**Table 1:** List of HcRNAV clones examined in the present study

Clone No.	Location	Date	Depth	Host	Reference
34	Ago Bay	2001/7/31	*B-1 m	HA92-1	Tomaru *et al.* (17)
109	Obama Bay	2000/9/12	0 m	HY9423	Tomaru *et al.* (17)
144	Imari Bay	2002/7/9	5 m	HCLG-1	
147	Ago Bay	2002/7/18	5 m	HCLG-1	
152	Imari Bay	2002/7/23	5 m	HU9433-P	
167	Ago Bay	2002/8/5	0.5 m	HU9433-P	
318	Imari Bay	2002/10/2	B-1 m	HU9433-P	
326	Ago Bay	2002/10/16	B-1 m	HCLG-1	
328	Imari Bay	2002/10/18	5 m	HCLG-1	
333	Ago Bay	2002/11/11	5 m	HU9433-P	
336	Ago Bay	2003/2/3	5 m	HU9433-P	
631	Kusuura Bay	2007/9/21	3.8 m	HU9433-P	
635	Kusuura Bay	2007/9/21	Sediment	HU9433-P	
636	Kusuura Bay	2007/9/21	Sediment	HCLG-1	
639	Kusuura Bay	2007/9/211	5 m	HU9433-P	
640	Kusuura Bay	2007/9/21	5 m	HCLG-1	
641	Kusuura Bay	2007/9/21	5 m	05HC05	
643	Kusuura Bay	2007/9/21	Sediment	HU9433-P	
644	Kusuura Bay	2007/9/21	Sediment	HCLG-1	
645	Kusuura Bay	2007/9/21	Sediment	05HC05	
647	Kusuura Bay	2007/10/2	Sediment	HU9433-P	
649	Kusuura Bay	2007/10/2	Sediment	05HC05	
650	Kusuura Bay	2007/10/2	Sediment	HU9433-P	
653	Kusuura Bay	2007/10/2	Sediment	05HC05	
654	Kusuura Bay	2007/10/2	2.7 m	05HC06	
655	Kusuura Bay	2007/10/2	Sediment	05HC06	
658	Kusuura Bay	2007/10/24	Sediment	05HC05	
659	Kusuura Bay	2007/10/24	Sediment	05HC06	
660	Kusuura Bay	2007/10/24	Sediment	HU9433-P	
662	Kusuura Bay	2007/10/24	Sediment	05HC05	
663	Kusuura Bay	2007/10/24	Sediment	05HC06	
664	Kusuura Bay	2007/11/7	Sediment	HU9433-P	
665	Kusuura Bay	2007/11/7	Sediment	HCLG-1	
667	Kusuura Bay	2007/11/7	Sediment	05HC06	
668	Kusuura Bay	2007/11/7	Sediment	HU9433-P	
669	Kusuura Bay	2007/11/7	Sediment	HCLG-1	
671	Kusuura Bay	2007/11/7	Sediment	05HC06	
672	Kusuura Bay	2007/11/28	Sediment	HU9433-P	
674	Kusuura Bay	2007/11/28	Sediment	05HC05	
676	Kusuura Bay	2007/11/28	Sediment	HU9433-P	
678	Kusuura Bay	2007/11/28	Sediment	05HC05	
679	Kusuura Bay	2007/11/28	Sediment	05HC06	
680	Kusuura Bay	2007/11/28	Sediment	HU9433-P	
683	Kusuura Bay	2007/12/26	Sediment	05HC06	
684	Kusuura Bay	2007/12/26	Sediment	HU9433-P	
686	Kusuura Bay	2007/12/26	Sediment	05HC05	
687	Kusuura Bay	2007/12/26	Sediment	05HC06	
VA23_ago	Ago Bay	2009/7/28	Sediment	HC20A23	
VA35_ago	Ago Bay	2009/7/28	Sediment	HC20A35	
09HcRVkam2	Lake Kamo	2009/11/19	0 m	HU9433-P	
09HcRVkam3	Lake Kamo	2009/11/19	0 m	05HC06	
09HcRVkam15	Lake Kamo	2009/11/19	0 m	05HC06	
09HcRVkam18	Lake Kamo	2009/11/19	0 m	05HC06	

* B-1 m refers to 1 m from the bottom on.

**Table 2:** Simplified cross-reactivity between typical host strains and virus clones

Host strain	Virus sensitivity*											

	HcRNAV34			HcRNAV109			HcRNAV641			HcRNAV659		
HU9433-P	+	+	+	−	−	−	+	+	+	+	+	+
HCLG-1	−	−	−	+	+	+	−	−	−	−	+	+
05HC05	+	+	+	−	−	−	+	+	+	+	+	+
05HC06	−	−	−	−	−	−	−	+	+	+	+	+

* Virus sensitivity was evaluated using an inoculation experiment, Northern hybridization, and number of abnormal cells, respectively (from left to right). Number of “+” indicates the intensity of reactivity.
