# Supplementary material for: High Interaction Variability of the Bivalve-Killing Dinoflagellate Heterocapsa circularisquama Strains and Their Single-Stranded RNA Virus HcRNAV Isolates
Source: Microbes Environ. 2012 Dec 27;28(1):112–9. doi: 10.1264/jsme2.ME12106 (PMC4070693; doi:10.1264/jsme2.ME12106)
Supplement: Supplementary file 1 [file 28_112_s1.pdf]

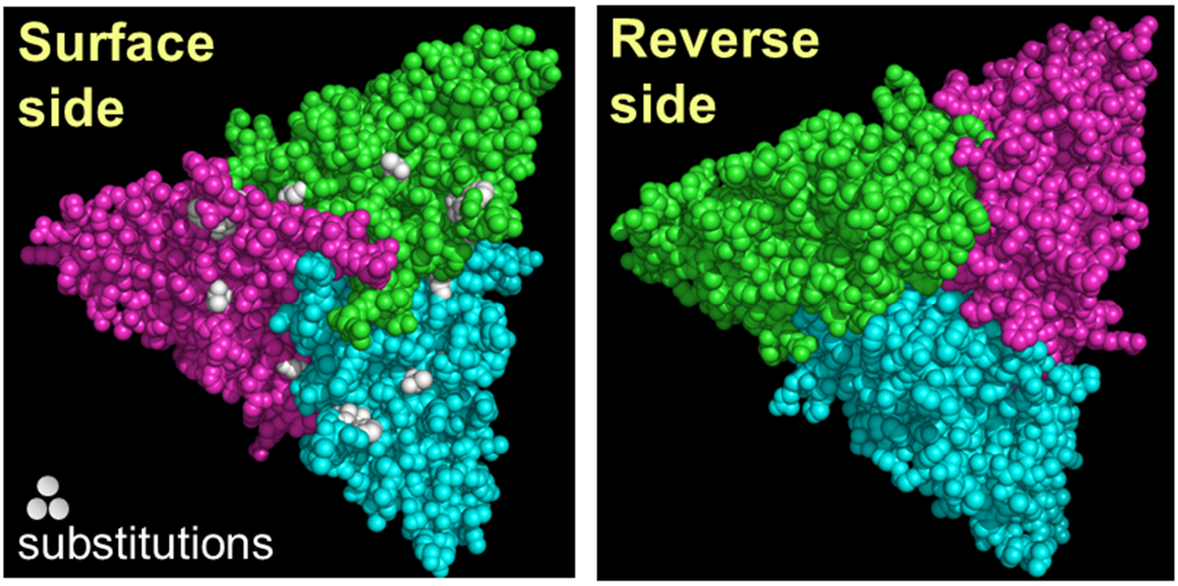

Suppl. Fig. S1. *In silico*-predicted ertiary structures of the major capsid trimer of HcRNAV659. Each monomer constructing the capsid trimer is differently colored, and the substituted amino acid residue in HcRNAV MCP (differing from HcRNAV34) is shown as white spheres.

Nakayama et al. Suppl. Fig. 1
